# Effects of Guanidinoacetic Acid Supplementation on Productive Performance, Pectoral Myopathies, and Meat Quality of Broiler Chickens

**DOI:** 10.3390/ani11113180

**Published:** 2021-11-07

**Authors:** Shady Khalil, Nualprae Saenbungkhor, Kajorn Kesnava, Panneepa Sivapirunthep, Ronachai Sitthigripong, Sukanya Jumanee, Chanporn Chaosap

**Affiliations:** 1 AlzChem Trostberg GmbH, 83308 Trostberg, Germany; Kajorn.Kesnava@extern.alzchem.com; 2 Behn Meyer Chemicals Co., Ltd., Bangkok 10520, Thailand; nualpraes@gmail.com; 3 Department of Agricultural Education, Faculty of Industrial Education and Technology, King Mongkut’s Institute of Technology Ladkrabang, Bangkok 10520, Thailand; panneepas@yahoo.com; 4 Department of Animal Production Technology and Fisheries, Faculty of Agricultural Technology, King Mongkut’s Institute of Technology Ladkrabang, Bangkok 10520, Thailand; ronachai.sit@kmitl.ac.th (R.S.); sukanya19837@gmail.com (S.J.)

**Keywords:** guanidinoacetic acid, creatine, glycogen, broiler, meat quality, wooden breast

## Abstract

**Simple Summary:**

Genetic selection for rapid growth is accompanied with challenges in meat quality such as pectoral myopathies, which lead to downgrading of breast muscle and economic losses for slaughterhouses. This experiment evaluated the effects of guanidinoacetic acid supplementation at rate of 0%, 0.06%, and 0.12% on the productive performance and meat quality of broiler chickens. Result showed that wooden breast was manifested by low creatine and high ultimate pH, and more associated with heavy birds. Guanidinoacetic acid supplementation increased muscle glycogen, reduced the ultimate pH, and reduced the incidence of wooden breast severity. In conclusion, guanidinoacetic acid can be used in broiler diets to improve the productive performance without exacerbating pectoral myopathy or affecting meat quality.

**Abstract:**

The effects of guanidinoacteic acid (GAA) supplementation on productive performance, pectoral myopathies, and meat quality of broilers were studied. Treatments consisted of corn/soybean-based diets with a GAA supplement (0%, 0.06%, and 0.12%). A total of 546 one-day-old Ross-308 males were randomly allocated to 42 floor pens with 14 replicates (13 birds/pens) for each treatment. The results showed that GAA at doses of 0.06% and 0.12% improved feed conversion, increased the percentage of normal breast, and decreased the severity of wooden breast. Breast muscle myopathy severity was positively correlated with heavy birds and negatively correlated with breast muscle creatine and glycogen. Breast muscle creatine and glycogen correlated positively with normal, less severe pectoral myopathies and meat quality. In conclusion, GAA supplementation improved broiler performance without exacerbating pectoral myopathy or affecting meat quality.

## 1. Introduction

Genetic selection for rapid growth combined with improvement in feed efficiency has led to affordable and sustainable meat production. Improvements in meat production, particularly in broilers, have been accompanied by challenges in meat quality, such as pectoral myopathies [1]. These myopathies lead to downgrading or condemnation of carcasses and economic losses for slaughterhouses as the breasts of these birds, which are considered premium cuts in the poultry industry, must be discarded due to their condition [2]. Deep pectoral myopathy was initially reported in turkeys in the 1960s in many countries and described as degenerative myopathy. In the 1980s, it was reported in meat-type chicken. The affected muscle was described as pinkish discoloration associated with hyperemia. Later, green and grey-green discoloration may be seen at the advanced lesions stage. The latter is associated with damaged blood vessels and necrosis, and is linked to ischemic necrosis triggered by rapid physical activity [3]. In modern broiler chickens, two major myopathies faced by the broiler industry are wooden breast (WB) and white striping (WS). WS is characterized by white striation of fat and connective tissues that run parallel to the myofibers in the breast meat [4]. Prevalence of WS has been reported to have increased from 5% in 2012 to over 90% in recent years [5,6]. WB is characterized by discoloration of the muscles, hard consistency, turbid, viscous coating on the outer surface, and reduced myofibrillar protein [4]. Both WS and WB have been reported to affect the water-holding capacity of meat, resulting in higher drip loss and cooking loss, and lower marinade absorption [7,8,9]. Moreover, the severity of WB and WS corresponded to higher pH and also higher lightness, redness and yellowness [10]. In addition, Del Campo et al. [10] reported that the hardness of raw meat increased with the severity of WB.

Pectoral muscle of the broiler chicken is almost completely white muscle fiber (type IIB, glycolytic muscle fiber), which is characterized by fewer blood capillaries and fewer mitochondria compared to the red muscle fiber. This can be especially problematic when the pectoral muscle is affected by hypoxia due to ischemia. Pathologically, muscle fiber can grow so rapidly at the expense of the supporting connective tissue space that carries the blood vessels, resulting in an ischemic condition [4,11]. Poor blood circulation in the enlarged pectoral muscle leads to accumulation of lactic acid and metabolic waste, low pH, loss of muscle creatine (Cre), and high serum creatine kinase (CK), resulting in less efficient recovery and muscle damage [4,12].

Cre is a metabolite that can be synthesized de novo from three amino acids: glycine (Gly), arginine (Arg), and methionine (Met) [13]. Cre plays a direct role in protein accretion, has been proposed as an indicator of meat quality [14], and has been shown to have a protective effect on the cell membrane [15]. In practice, it is possible to increase Cre by increasing the arginine: lysine (Arg:Lys) ratio, but only up to a certain limit, otherwise performance may be compromised due to Arg-Lys antagonism [16]. Feeds of animal origin have a significant but highly variable Cre content which is negatively affected by the rendering process [13,17,18].

Guanidinoacetic acid (GAA) is the natural precursor of Cre. GAA is methylated by S-adenosylmethionine to form Cre in a reaction catalyzed by S-adenosyl-L-methionine: N-guanidinoacetate methyltransferase [14]. GAA showed better stability during feed processing and storage compared to Cre [19]. Previous studies have shown that GAA supplementation spared Arg in broiler diets and more Cre was present in the breast muscles than in the Arg supplemented group. This may suggest that GAA is a better candidate for mitigating breast myopathies because it can increase the availability of Arg and Cre simultaneously without affecting broiler performance [20].

Many studies have shown the positive effect of GAA on production performance [13,14,17]; however, there are limited data on the effect of GAA on pectoral myopathies in modern intensive broiler production. In this study, it was hypothesized that GAA could improve the production performance of broiler chickens raised at high stocking density while alleviating breast muscle myopathies and improving meat quality. Therefore, the current study was conducted to investigate the effects of dietary supplementation with GAA at doses of 0.06% and 0.12% on production performance, carcass characteristics, yield, wooden breast, and white striping in broiler chickens raised at high stocking density, and to investigate the effects of myopathies on meat quality.

## 2. Materials and Methods

### 2.1. Treatments and Husbandry of Birds

The handling and care of animals in research was approved by the Institutional Animal Care and Use Committee at King Mongkut’s Institute of Technology Ladkrabang (KMITL), Bangkok, Thailand (AUAC-KMITL-RES/2020/007). The birds were raised and slaughtered at the Bangkok Animal Research Center Co., Ltd. (BARC), Samutprakan, Thailand. Five hundred and forty-six newly hatched male chicks of a commercial strain (Ross 308) were divided into 3 treatments, each treatment containing 14 replicates as an experimental unit. Treatment one (T1) was fed a basal diet and served as a control group, while treatments two (T2) and three (T3) were fed a basal diet supplemented with 0.06% and 0.12% guanidinoacetic acid (GAA) (Creamino^®^, at least 96% GAA; AlzChem Trostberg GmbH, Trostberg, Germany), respectively. The experiment was conducted in a closed house with tunnel ventilation and evaporative cooling. Birds were reared in houses with solid concrete floors and rice husks as bedding material. Stocking density was 13 birds/m^2^ (41 kg/m^2^ at 42 days of age) and was equipped with a tube feeder and three nipple drinkers. Feed and water were provided ad libitum. Birds were exposed to a continuous light program of 24L:0D (40 lux light intensity) for the first 7 days of age. Day length was gradually reduced to 20L:4D (10 lux) until 42 days of age. The average brooding temperature was 33.0 °C with 34.74 °C Max and 28.18 °C Min with a relative humidity of 74.81% and air velocity of 7.6–15.2 m/min during the first 3 days, followed by 15.2–22.9 m/min up to 7 days. After brooding, from 7 to 10 days, the average temperature was 31 °C with 33.17 °C Max and 25.70 °C Min with a relative humidity of 76.38% and air velocity of 15.2–22.9 m/min. At 10–28 days of age, the average temperature was 28.5 °C with 30.78 °C Max and 26.17 °C Min with a relative humidity of 82.58% and an air velocity of 8.2–8.5 m/min. At 29–42 days of age, the average temperature was 27.5 °C with 30.31 °C Max and 25.30 °C Min with a relative humidity of 83.81% and an air velocity of 8.2–8.5 m/min. All birds were vaccinated against Newcastle and infectious bronchitis diseases at 7 days of age and against Gumboro disease at 14 days of age.

### 2.2. Diets

A practical corn/soybean-based diet was formulated to meet the nutritional recommendations of Ross 308 [21] and was used as a basal diet for each growth stage (Table 1). Starter, grower, and finisher diets were fed at 0–10, 10–28, and 28–42 days of age, respectively. All diets were mixed using a horizontal double ribbon mixer. Mash diets were processed at a conditioning temperature of 82 °C and pellet size of 3 mm diameter. For the first 10 days, the diets were offered to the birds in crumbled form and then in pelleted form until the completion of the 42-day test period. After crumbling or pelleting, representative samples of each diet produced were analyzed for crude protein and total amino acids. The GAA content of the diet was determined by AlzChem AG (Trostberg, Germany) using the ion chromatography method [22].

### 2.3. Growth Performance

Body weight (BW) was measured on days 10, 28, and 42 of age. Body weight gain (WG), feed intake (FI) and feed conversion ratio (FCR) were calculated for the period of 0–10, 0–28, and 0–42 days of age.

### 2.4. Carcass, Cutting Yield, and Muscle Sampling

On day 42, 4 birds per pen were randomly selected in all treatments and fasted overnight, weighed, and then slaughtered in BARC’s slaughter unit. Birds were stunned by CO_2_ before exsanguination, scalded at 58–60 °C for 120 s, de-feathered with a rotating drum picker for 2 min, and manually eviscerated. Carcasses were then chilled in ice water for 15 min, kept in a cold room at 4 °C for at least 30 min until core temperature less than 7 °C to separate water residues from the chilling process, and then weighed. The whole carcass was dissected manually with a knife and the weights of all dissected parts; breast, fillet, wing, and leg were documented as a percentage of the total carcass weight.

**Figure 1:**
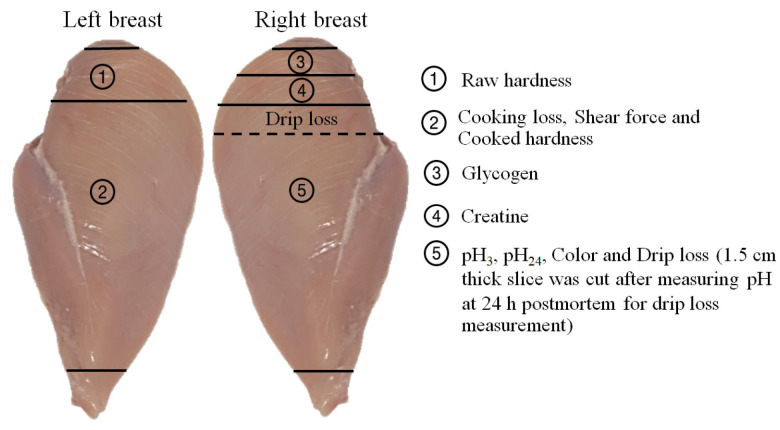
Pectoralis major muscle sampling location for the evaluation of glycogen content, creatine content, and meat quality.

After dissection, the left pectoral muscles were used to evaluate muscle myopathies and were then vacuum packed and stored at −20 °C for approximately 2 weeks before cooking loss, shear force, and hardness were evaluated (Figure 1). The right pectoral muscle was cut from the upper cranial part to obtain two pieces of a 50 g meat sample for the muscle, which were snap frozen in liquid nitrogen and stored at −80 °C to be analyzed for creatine and glycogen content. The remaining breast muscle was used to measure pH_3_ and meat color 3 h postmortem before being stored at approximately 1 °C for up to 24 h postmortem before measuring pH_24_ and drip loss.

### 2.5. Pectoral Myopathies

**Figure 2:**
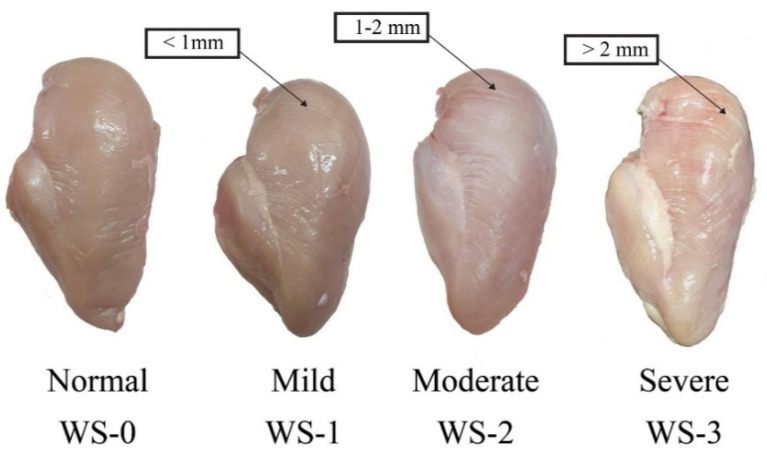
Visual scoring scale for white striping (WS) in pectoral muscle where 0 = normal, 1 = mild, 2 = moderate, and 3 = severe. Normal—No distinct white lines. Mild—Small white lines, generally <1 mm thick, but apparently visible on the breast surface. Moderate—Large white lines (1–2 mm thick) very visible on the breast surface. Severe—Thick white bands (>2 mm thickness) covering almost entire surface of breast.

**Figure 3:**
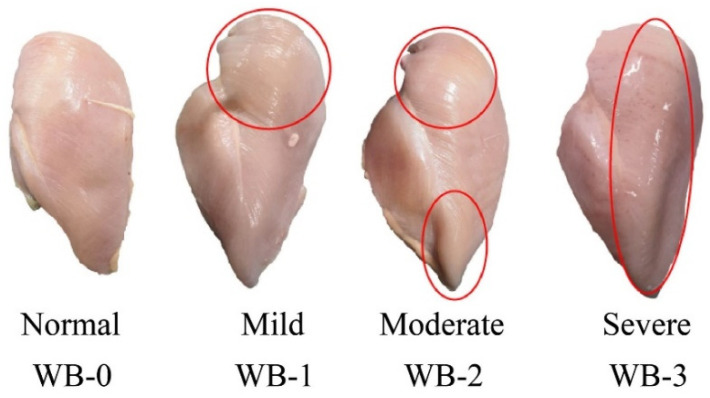
Wooden breast (WB) scoring scale based on tactile assessment where score 0 = normal breast muscle which is flexible throughout; score 1 = mild breast muscle, which is hard mainly in the cranial region but flexible elsewhere; score 2 = moderate breast muscle, which is hard throughout but flexible in the mid to caudal region; score 3 = severe muscle, which is extremely hard and rigid from the cranial region to the caudal tip.

A total of 168 (4 samples/pen from each treatment) breast samples were trimmed to remove cartilage, visible fat, and connective tissue for WB and WS evaluation. Left pectoral muscle was classified for WS according to Kuttappan et al. [23] with slightly modification. WS breast muscle was scored according to the thickness and intensity of white striation where: score 0 = normal when there is no white striation; score 1 = mild when breast muscle has small thin white lines (<1 mm); score 2 = moderate, when breast muscle has large white striations (1–2 mm) that are easily visible on the fillet surface; score 3 = severe when a thick white band (>2 mm) covers almost the entire surface of the fillet (Figure 2). In addition, the left pectoral muscle was also individually evaluated for WB based on tactile assessment according to Tijare et al. [24] and scored as follows: score 0 = normal breast muscle which is flexible throughout; score 1 = mild breast muscle, which is hard mainly in the cranial region but flexible elsewhere; score 2 = moderate breast muscle, which is hard throughout but flexible in the mid to caudal region; score 3 = severe muscle, which is extremely hard and rigid from the cranial region to the caudal tip (Figure 3).

### 2.6. Creatine and Glycogen Evaluation

For creatine and glycogen analysis, a 50 g meat sample was taken at 2 cm from the upper cranial part of the right breast muscle sample from each myopathy score. Creatine concentration was determined by Swiss BioQuant AG (Reinach, Switzerland) as follows: Creatine reference substance (Sigma-Aldrich, St. Louis, MO, USA). A HPLC-MS /MS system (Agilent 1200 series pump, HTS PAL autosampler, and Thermo Fisher Scientific TSQ Vantage mass spectrometer) was used for creatine analyses, Reprosphere HILIC-A trapping column (10 mm × 2 mm ID, 5 µm) (Dr. Maisch HPLC GmbH, Ammerbuch, Germany), and iHILIC Fusion analytical column, 20 mm × 2.1 mm ID, 5 µm (HILICON AB, Umea, Sweden). Approximately 50 mg of tissue was placed in a homogenization tube and the exact weight was determined. Nine volume equivalents of water were added to a homogenization tube (e.g., 450 µL of water was added to 50 mg of tissue). The sample was homogenized for 30 s at 6500 rpm. Depending on the type of tissue, this may be repeated until the sample is sufficiently homogenized. After homogenization, the sample was centrifuged at 16,000× *g* for 10 min and the supernatant was used for further analysis. If necessary, the homogenates were further diluted with water to a concentration within the calibration range. Creatine was determined by column separation with hydrophilic interaction liquid chromatography followed by detection with a three-step quadrupole MS/MS in selected reaction monitoring mode (SRM) under the following conditions: Trap column: Dr. Maisch Reprosphere HILIC-A, 10 mm × 2 mm ID, 5 µm Analytical column: iHILIC Fusion, 20 mm × 2.1 mm ID, 5 µm Eluent: water containing 10 mM ammonia acetate with 0.05% formic acid and acetonitrile. Injection volume: 50 µL. Flow rate: 0.5 mL/min. Column temperature: 40 °C. Detection: SRM in positive mode. Glycogen content in each muscle sample was determined following the protocol of Dreiling et al. [25] with slight modifications according to Chaosap et al. [26].

### 2.7. Meat Quality Evaluation

#### 2.7.1. pH Measurement

The pH was measured in duplicate at 3 and 24 h postmortem directly on the right pectoralis major muscle using a pH meter with a spear glass electrode, model SG2-ELK Seven GoTM, Mettler Toledo International Inc. Giessen, Germany.

#### 2.7.2. Color Measurement

The color of the right pectoralis major muscle was measured after 45 min of bloom time, CIE color values L* (lightness), a* (redness), and b* (yellowness) were determined at two different locations on the muscle surface using a spectrophotometer with an aperture of 2.54 cm (MiniScan EZ 45/0 LAV, illuminance D65, 10° observer, Hunter Associates Laboratory Inc, Reston, VA, USA).

#### 2.7.3. Drip Loss Measurement

To determine drip loss, each 1.5-cm-thick sample of right pectoralis major muscle was weighed 3 h postmortem and immediately placed in a plastic bag, suspended from a hook, and stored for 48 h at 4 °C. After hanging, the sample was carefully wiped with a paper towel and weighed again. The weight difference, which corresponded to the drip loss, was expressed as a percentage of the original muscle weight.

#### 2.7.4. Cooking Loss, Shear Force and Hardness Measurement

For cooking loss, each left pectoralis major muscle was weighed 24 h postmortem and then cooked in vacuum-sealed bags in a water bath at 80 °C until an internal temperature of 70 °C was reached. The meat sample was cooled under running tap water for 30 min and weighed again. The difference in weight of the cooked sample was expressed as percentage of cooking loss. Each cooked meat sample was sliced parallel to the fiber orientation to obtain as many 1 cm × 2 cm × 1 cm wide × long × thick slices as possible. Each slice was sheared once perpendicular to the muscle fiber orientation using a Warner-Bratzler shearing head attached to a single-column Texture Analyzer Machine (model EZ-SX, Shimadzu, Tokyo, Japan) with a 50 kg load cell and a crosshead speed of 50 mm/min. Raw and cooked muscles were cut into three 1.5 cm × 1.5 cm × 1.5 cm wide × long × thick slices parallel to the fiber orientation. A Texture Analyzer Machine (Model EZ-SX, Shimadzu, Tokyo, Japan) was used to determine the hardness of the specimens. The specimens were placed under a cylindrical probe with a diameter of 36 mm and the probe descended at a constant speed of 127 mm/min.

### 2.8. Statistical Analysis

The effect of GAA levels on growth performance, carcass and meat quality characteristics were analyzed using a general linear model with Proc GLM (SAS Institute Inc., Cary, NC, USA). Least squares means were separated using the PDIFF option. The chi-square test of independence and the proportion Z test were used to determine whether the distribution frequency (*n*) and percent severity of breast myopathies at 42 days of age were caused by the different GAA supplementation levels (SPSS version 21, SPSS Inc, Chicago, IL, USA). To evaluate the effects of the WB and WS categories on the different parameters, Levene’s test was first used to test for equality of error variances, and Kolmogorov-Smirnov was used to test for normality of parameter residuals. The effect of unequal groups of WB and WS categories with equal variances on carcass and meat quality characteristics were analyzed using a general linear model with Proc GLM, where WB and WS were used as the main effect and due to the interaction between WB and WS was not significant therefore the interaction effect was removed from the statistical model. The least squares means were compared using the PDIFF option in SAS and the significance level was at a *p*-value of less than 0.05. Principal component analysis (PCA) of the studied parameters was analyzed using the XLSTAT software (Addinsoft, New York, NY, USA).

## 3. Results

### 3.1. Diets

The analyzed crude protein and total amino acids were homogenous among groups with a coefficient of variation (CV, %) less than 5% and more or less equal to the calculated values as shown in Table 1. Creamino^®^ (product basis) and GAA (active substance) showed a good recovery in the final feed compared to the calculated values as shown in Table 2.

### 3.2. Effect of Guanidinoacetic Acid Supplementation on Growth Performance

Results of growth performance are shown in Table 3. No significant difference was observed in BW and cumulative WG at all growth phases (*p* > 0.05). GAA supplementation at 0.06% and 0.12% significantly reduced cumulative FI at 28 days of age compared to the control group (*p* < 0.05). At 10 days of age, GAA supplementation at 0.06% and 0.12% significantly decreased cumulative FCR by 2.09% and 3.15%, respectively, and at 42 days of age by 3.14% and 3.39%, respectively, compared to the control group (*p* < 0.05).

### 3.3. Effect of Guanidinoacetic Acid Supplementation on Carcass Composition and Carcass Cuts

Live weight, carcass weight, and carcass yield were not affected by dietary GAA level. There were no significant differences among the treatments in weight and relative percentage of all cuts Table 4.

### 3.4. Effect of Guanidinoacetic Acid Supplementation on Pectoral Myopathies

The distribution of probability for each WB and WS score at 42 days of age is shown in Table 5. The group supplemented with 0.12% GAA showed a significant reduction in WB score 3 by 20% difference compared to the control group (*p* < 0.05). The latter attenuation of the WB severity score resulted in a higher proportional distribution of lower WB severity scores compared to the control group but was not sufficient to show significance (*p* > 0.05). The 0.06% GAA supplemented group reduced WB score 3 by 11% difference compared to the control group, which resulted in a higher proportional distribution of lower WB severity scores but was not sufficient to reach the significance level (*p* > 0.05). On the other hand, WS affected breast muscle showed no significant difference among the groups (*p* > 0.05). Interestingly, the incidence of WS score 3 was very low, with only one case in the control group and none in the GAA supplemented groups. Nevertheless, the group supplemented with 0.12% GAA showed a 10% reduction difference in score 2 compared to the control group and 5% in the group supplemented with 0.06% GAA compared to the control group.

### 3.5. Effect of Guanidinoacetic Acid Supplementation on Meat Quality

The results showed that the level of dietary GAA had no effect on muscle Cre (*p* > 0.05) as shown in Table 6. The glycogen content in the pectoral muscle was significantly increased by 332% and 382% in the groups with 0.06% and 0.12% GAA, respectively, compared to the group without GAA supplementation (*p* < 0.05). The effect of glycogen content on pectoral muscle pH was even more significant. The pH of the pectoral muscle was lower with GAA supplementation of 0.06% and 0.12% compared to the control group (*p* < 0.05). There were no significant differences in meat color, drip loss, cooking loss, and shear force (*p* > 0.05).

### 3.6. Effect of Myopathies on Carcass Composition

Live weight and carcass weight increased with the presence of WS from 3.10 kg to 3.23 kg and from 2.56 kg to 2.65 kg, respectively (*p* < 0.1). Nevertheless, the presence of WB was not associated with live weight, carcass weights, and carcass yield (*p* > 0.05). The presence and severity of WB score 2 was specifically associated with higher breast weight, fillet weight, breast yield and fillet yield (*p* < 0.05). On the other hand, the presence of WS was not associated with differences in above parameters (*p* > 0.05) Table 7.

### 3.7. Effect of Myopathies on Creatine Content, Glycogen Content, and Meat Quality

The breast muscle Cre decreased from 5206.11 to 3894.71 mg/kg with the presence of WB severity, especially WB score 2 and 3 (*p* < 0.05). The presence of WB was not significantly associated with glycogen and pH_3_ (*p* > 0.05), but WB score 3 was significantly associated with higher pH_24_ (*p* < 0.05). On the other hand, the presence of WS was not associated with glycogen, pH_3_ and pH_24_ (*p* > 0.05). Moreover, WB severity was significantly associated with higher L* and b* but not a*. Nevertheless, the occurrence of WS was not associated with meat color (*p* > 0.05). Higher drip loss was strongly associated with WS score 1 (*p* < 0.05) but not with cooking loss (*p* > 0.05). On the other hand, higher cooking loss was strongly associated with WB severity scores (*p* < 0.05), but not with drip loss (*p* > 0.05). Shear force decreased from 4.52 kg to 3.54 kg with the appearance of WB severity (*p* < 0.05). In contrast, shear force was not associated with the occurrence of WS (*p* > 0.05). WB score 3 was strongly associated with higher raw meat hardness (*p* < 0.05), while WS score 2 was strongly associated with higher cooked meat hardness (*p* < 0.05) (Table 8).

### 3.8. Principal Component Analysis

**Figure 4:**
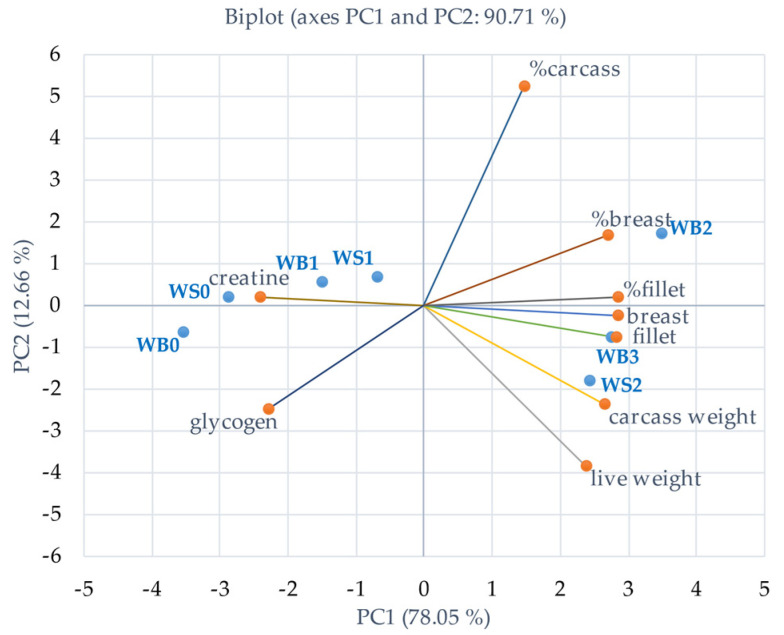
Principal component analysis biplot of wooden breast (WB) and white stripping (WS) severity score and carcass composition of broiler chickens at 42 days of age. WB; 0, 1, 2, 3 = wooden breast scores are based on a 4-point scale (3 = severe, 2 = moderate, 1 = mild, and 0 = normal); WS; 0, 1, 2, 3 = white striping scores are based on a 4-point scale (3 = severe, 2 = moderate, 1 = mild and 0 = normal).

Principal component analysis (PCA) was performed to evaluate the relationships between carcass composition variables and myopathy severity. The PCA biplot of carcass composition data is shown in Figure 4. PC1 and PC2 explained 90.71% of the total variance. The first PC (78.05% of total variance) was positively loaded by live weight, carcass weight, fillet weight, breast weight, %breast, and %fillet but negatively loaded by Cre and glycogen content. The second PC (12.66% of total variance) was positively loaded by %carcass. In the biplot, WS score 2, WB score 3, and WB score 2 were closely associated with live weight, carcass weight, breast weight, fillet weight, %breast, and %fillet. Interestingly, breast muscle Cre and glycogen content is on the opposite side of WB score 2 and score 3 and WS score 2 but is more closely associated with normal or less severe breast muscle myopathies.

**Figure 5:**
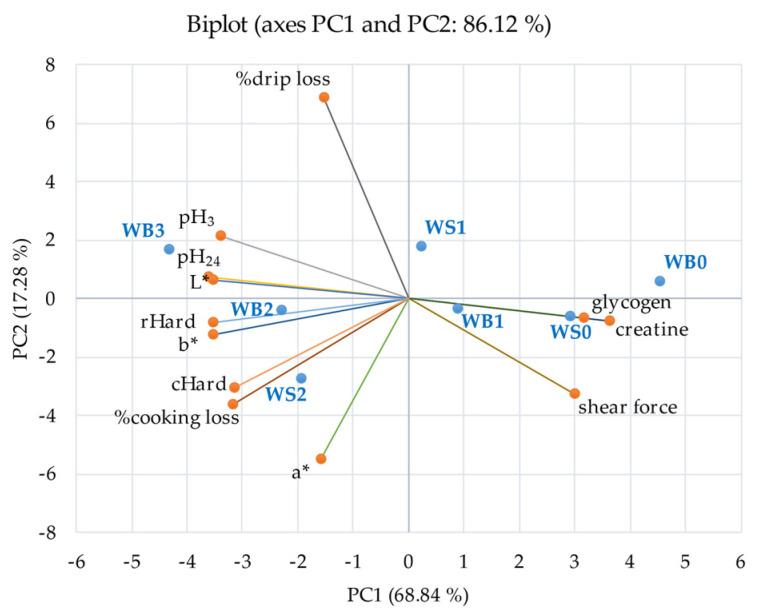
Principal component analysis biplot of the wooden breast (WB) and white stripping (WS) severity score and meat quality attributes of broiler chickens at 42 days of age. WB, 0, 1, 2, 3 = wooden breast scores are based on a 4-point scale (3 = severe, 2 = moderate, 1 = mild, and 0 = normal); WS, 0, 1, 2, 3 = white striping scores are based on a 4-point scale (3 = severe, 2 = moderate, 1 = mild, and 0 = normal). cHard = cooked meat hardness, rHard = raw meat hardness, L* = lightness, a* = redness, and b* = yellowness.

The PCA biplot of meat quality data is shown in Figure 5. PC1 and PC2 explained 86.12% of the total variance. PC1 (68.84% of total variance) was positively loaded by shear force, muscle Cre, and glycogen content but negatively loaded by cooking loss, cooked meat hardness, raw meat hardness, L*, b*, pH_3_, pH_24_, and drip loss. On the other hand, PC2 (17.28% of total variance) was negatively loaded by a*, but positively loaded by % drip loss. From the bi-plot, severe myopathy scores were closely related to pH_3_, pH_24_, L*, b*, cooking loss, drip loss, cooked, and raw hardness. While normal and mild myopathy (WB and WS) of pectoral muscle on the opposite side of more severe myopathy of pectoral muscle were closely related to shear force and muscle Cre and glycogen content.

## 4. Discussion

### 4.1. Effect of Guanidinoacetic Acid Supplementation on Growth Performance

Dietary GAA supplementation was used as a possible nutritional approach to attenuate pectoral myopathies. Birds in all groups were able to reach the maximum growth potential of the male Ross-308 breed [27] at a high stocking density of 13 birds/m^2^ (40.8–41.5 kg/m^2^ at 42 days of age). Nevertheless, the GAA supplemented groups (0.06% GAA and 0.12% GAA) were able to significantly reduce cumulative FI at 28 days of age and cumulative FCR at day 10 and 42, while maintaining BW. Previous studies have confirmed the effect of GAA supplementation on growth performance. Córdova-Noboa et al. [28] reported that 0.06% GAA supplementation resulted in 2.44% reduction in FCR from 0 to 50 days of age, regardless of the effect of grain type. The same authors reported improvements in BW and WG at 14 and 35 days of age, but not at 50 days of age. Amiri et al. [29] recorded a 4.52% and 5.65% reduction in FCR at 0 to 42 days of age in the groups receiving 0.06% and 0.12% GAA as the main effect, respectively, regardless of the effect of the basal diet formulated with normal or low protein compared to the non-supplemented groups. Moreover, GAA as main effect improved average daily feed intake (ADFI) at 0 to 10, 24 to 42, and 0 to 42 days of age. In addition, BW and WG improved at 42 days of age. Boney et al. [17] recorded a 2.69% reduction in FCR from day 1 to 21 of age and a 2.55% reduction in FCR from day 36 to 42 of age in the diets supplemented with 0.06% GAA, regardless of the effect of the basal diet (conventional protein source or non-protein source), while BW was improved at 21 days of age compared to the non-supplemented groups. Khalil et al. [13] reported that GAA at the level of 0.06% as the main effect on FCR resulted in a 4.03% reduction, regardless of the effect of dietary T3—hormone compared to the groups without GAA supplementation. Our results can be explained by the fact that GAA could improve the efficiency of energy utilization by replenishing ATP through the Cre-PCre shuttle system in addition to its Arg-sparing effect, which plays a central role in endogenous nitric oxide synthesis and maximizing growth performance of broiler chickens [14].

### 4.2. Effect of Guanidinoacetic Acid Supplementation on Carcass Composition and Carcass Cuts

In our study, carcass composition and carcass cuts did not differ significantly between groups. In agreement with our results, it was found that the group receiving 0.06% GAA to a basal vegetarian diet with different energy levels until 41 days of age had no significant effect on carcass parameters in male broiler chickens compared to non-supplemented groups [30]. The same authors reported in second experiment that 0.06% GAA supplementation to poultry by-product meal basal diets with different energy levels had no significant effects on carcass composition compared to the non-supplemented groups up to 41 days of age. Khalil et al. [13] reported that carcass and cutting yield was not affected by 0.06% GAA supplementation compared to the non-supplemented group. However, another study showed a significant increase in both breast weight and yield in groups supplemented with 0.06% GAA as the main effect in basal diets with or without animal protein source compared to groups without GAA supplementation, while other carcass traits and relative organ weights were unaffected [17]. Further studies are needed to highlight the effect of GAA supplementation on carcass composition in modern broiler chickens so that the data are more accurate and can be better interpreted.

### 4.3. Effect of Guanidinoacetic Acid Supplementation on Pectoral Myopathies

Our results showed that the GAA supplemented groups had the largest normal pectoral muscle and the lowest WB scores compared to the control group. However, WS showed no significant difference among the groups. Previous studies have shown that GAA supplementation reduces the occurrence of WB. Córdova-Noboa et al. [28] reported that GAA supplementation at 0.06% could reduce the severity of WB by increasing the number of breast muscles with low WB scores, but no significant effect of GAA supplementation on WS was observed. In addition, [31] reported that an improvement in WB severity at 56 days of age was observed by 0.06% GAA supplementation in the basal diet without poultry by-products compared to the control group. The latter showed a significant increase in the number of pectoral muscles with a low WB score to almost double and a lower WB severity score. To our knowledge, no study has investigated the effect of GAA on WB or WS with a higher percentage of GAA (0.12%). Therefore, our results, as well as previous findings may more strongly support the efficacy of GAA supplementation in attenuating WB severity than WS.

### 4.4. Effect of Guanidinoacetic Acid Supplementation on Meat Quality

GAA supplementation resulted in a significant increase in breast muscle glycogen, which in turn resulted in a significant decrease in pH_3_ and pH_24_ in breast muscle compared to the control group. Cre and other meat quality parameters did not differ among groups in the current study. In agreement with our results, Degroot et al. [32] found that free Cre in breast muscle was not significantly affected by dietary GAA supplementation at 0.06% and 0.12%, but glycogen, phosphocreatine (PCre), and total Cre were significantly increased compared to the negative Arg deficient control group. Moreover, [20] reported in the first study that free Cre and glycogen were significantly increased in the 0.18% GAA supplemented group, while PCre was significantly increased in the 0.12% and 0.18% GAA supplemented group compared to the Arg-deficient-negative control group. In the second study by the same authors, they reported that free Cre and PCre in breast muscle were not significantly increased by 0.06% and 0.12% GAA supplementation, but total Cre and glycogen were numerically increased in the 0.12% GAA group compared with GAA-devoid Arg-adequate control group. Zhang et al. [33] demonstrated that GAA supplementation at 0.12% significantly maintained muscle glycogen at a high level after the 3-h transport before slaughter compared to the group without GAA supplementation (*p* < 0.05). Previous studies reported that GAA supplementation at 0.06% had no effect on postmortem pH_6_ and pH_24_ in broilers fed corn-based diets with or without poultry by-products [31]. However, [28] indicated that in corn or sorghum-based diets, post mortem pH at 24 h was significantly lower in groups supplemented with 0.06% GAA than in group without GAA. On the other hand, the same study reported that at 55 days of age, postmortem pH_6_ and pH_24_ were reduced in groups supplemented with GAA compared to groups not supplemented with GAA (*p* < 0.05). Michiels et al. [34] reported that GAA at 0.06% and 0.12% significantly increased L* and b* compared to a vegetarian-based diet, but not significantly compared to a diet containing animal protein. In agreement with our study, [31] reported no change in meat color as a result of GAA supplementation as the main effect in diets with or without poultry by-products. Moreover, a previous study showed that GAA supplementation at 0.06% and 0.12% significantly reduced both cooking and drip loss compared to the non-supplemented group during pre-slaughter transport stress [33]. However, other studies reported that GAA supplementation had no effect on cooking loss and drip loss [28,31,34]. In agreement with our study, several studies reported that shear force was not affected by GAA supplementation [28,31]. The effects of GAA supplementation on glycogen and pH in our study reflect the crucial role of the phosphagen system in maintaining energy homeostasis in the pectoral muscle. It is worth mentioning that about 60–70% of total muscle Cre is in the form of PCre [35], which can prevent glycogen depletion and muscle damage [31]. Therefore, the glycogen content in the pectoral muscle can be used as a strong indicator of PCre and the energetic state of the pectoral muscle. The latter may explain the significant increase in pectoral muscle glycogen content, although Cre was numerically increased by 6% and 11% in the group with 0.06% and 0.12% GAA, respectively. Postmortem meat pH is influenced by glycogen content in the pectoral muscle [34], which explains the significantly low pH_3_ and pH_24_ in our study.

### 4.5. Effect of Myopathies on Carcass Composition

Our data showed that higher breast weight, fillet weight and their yields were strongly related to WB severity, but WS severity tended to be associated with live and carcass weight. Previous studies indicated that WB and WS were associated with breast weight [8], carcass weight and breast yield [10]. In addition, Kuttappan et al. [36] reported that severe WS was strongly associated with body weight, fillet weight, and yield in broilers.

### 4.6. Effect of Myopathies on Creatine, Glycogen Concentrations and Meat Quality

In the current study, it was clear that muscle myopathies were associated with a reduction in Cre, but this was more pronounced in WB than in WS. There is little or no data that has measured Cre and glycogen levels for each myopathy score.

Regarding pectoral muscle pH for different myopathy scores, our study did not agree with previous studies showing that moderately WS affected pectoral muscles had higher pH [37,38,39]. However, in agreement with our study, [40] reported that the pH of WS (with moderate to thick white striation) affected muscle was not significantly different compared to the pH of normal pectoral muscle (*p* > 0.05). Severely WB affected pectoral muscles showed higher pH compared to normal pectoral muscles [5,41,42]. In contrast, moderately WB affected pectoral muscle showed no difference (*p* > 0.05) in pH compared to normal pectoral muscle [42,43].

Regarding meat color, in agreement with our studies, [43,44] reported an increase in L* and b* in severely WB affected pectoral muscle (*p* < 0.05), while a* was not affected in moderately [43] and severely [8] WB affected muscle (*p* > 0.05). On the other hand, several studies reported no changes in L* in moderately [43] and severely [8,41] WB affected muscles compared to normal breast muscle (*p* > 0.05). Meanwhile, [41] showed that b* was not changed in severely WB affected muscles (*p* > 0.05). Moreover, a* was found to be increased in severe WB compared to normal pectoral muscle (*p* < 0.05) [41,43,44]. In agreement with our study, several studies showed no effects of moderate and severe WS on L*, b*, and a* (*p* > 0.05) [38,39,45,46,47]. Nevertheless, [45] reported that a* was significantly increased in moderately and severely WS affected pectoral muscles (*p* < 0.05).

Cooking and drip losses in our study were more or less consistent with other studies. It was reported that moderately [42] and severely [8,41,42,48] WB affected pectoral muscles had higher cooking loss compared to normal pectoral muscles (*p* < 0.05). In addition, moderately [45] and severely [37,45,49] WS affected pectoral muscles had higher cooking loss compared to normal pectoral muscles (*p* < 0.05). However, other studies reported no changes in cooking loss in moderately and severely WS affected pectoral muscles (*p* > 0.05) [37,38,46,47]. In terms of drip loss, [42] reported that moderately and severely WB had no effect on drip loss (*p* > 0.05), while many studies reported that severely WB affected pectoral muscle increased drip loss compared to normal pectoral muscle (*p* < 0.05) [5,8,41,44,48]. On the other hand, moderately [37,45] and severely [5,45,46] WS muscles showed no effect on drip loss (*p* > 0.05). In contrast, Alnahhas et al. [37] reported that severe WS had significantly higher drip loss (*p* < 0.05).

Regarding shear force, in agreement with our results, previous studies have shown that shear force was not affected in moderately [37,45,46,47] and severely WS affected pectoral muscles [46,47]. Although our results are not consistent with those of [42], who reported that shear force was not affected in moderately and severely WB [42,44], another study showed that shear force was significantly lower in severely woody breast (*p* < 0.05), which is consistent with our study [11,50].

In agreement with our study, previous studies showed a significant increase in raw meat hardness of severely WB affected meat (*p* < 0.05), while cooked meat hardness was not affected (*p* > 0.05) by WB severity [10,51]. On the other hand, the severity of WS, which affected raw and cooked meat, was not significantly affected (*p* > 0.05) [10]. In contrast, Zhang et al. [52] recorded a significant decrease in the degree of meat hardness of the batter gels of severely affected WB (*p* < 0.05).

### 4.7. Principal Component Analysis

Taking all variables together, severe myopathy scores were associated with heavier birds and manifested by low breast muscle Cre and glycogen Figure 4. Low breast muscle Cre and glycogen levels were strongly associated with undesirable meat quality parameters as shown in Figure 5. WB myopathy has been reported to be associated with hypoxia as a result of rapid growth, a reduction in glycolytic potential, impaired ATP production and mitochondrial damage [11]. Glycogen is a substrate of the glycolytic pathway (ante and postmortem) in the pectoral muscle, which may be depleted antemortem, leaving a low glycogen residue postmortem, resulting in a high ultimate pH postmortem [53]. The percentage of decrease of Cre in the weighted averages WB was −17% and −4% in WS and the percentage decrease of glycogen in the weighted averages WB was −31% and −26% in WS compared to normal meat. The latter may underline the importance of maintaining Cre and glycogen at high levels in modern broilers. High muscle Cre content has a protective effect by attenuating muscle cell damage, slowing the glycolytic process, and accelerating the conversion of lactic acid to glycogen by the liver. In addition, PCre was found to act as the major metabolic buffer in muscle by absorbing H^+^ during the ATP replenishment process in the Cre-PCre reaction [15]. It has been shown that Cre and glycogen are closely related in PCA and are on the other side of pH. Practically, Cre in breast muscle can be increased by dietary supplementation with GAA. Notwithstanding the fact that our experimental basal diets adequately met the Arg requirements [21], the Arg-sparing effect of GAA [14,18,20] may contribute to the reduction of WB through the action of nitric oxide [54]. The latter is a potent vasodilator that relaxes vascular smooth muscle, leading to better oxygenation of the pectoral muscle, which allows better removal of waste products and better recovery [55,56]. Although nitric oxide was not measured in our study, a previous study reported that high intake of GAA (0.075% and 0.15%) increased serum nitric oxide [56]. Apparently, GAA supplementation helped to attenuate the severity of WB incidence.

## 5. Conclusions

WB affected breast muscle was indicated by low Cre content, high ultimate pH, and poor meat quality such as paler color, higher cooking loss, higher raw hardness, and was more associated with heavy birds. High glycogen and Cre content in breast muscle were associated with normal, less severe myopathies and better meat quality. Overall, GAA as a precursor of Cre can be used as an effective feed additive in intensive rearing system for sustainable development as it could improve feed efficiency and reduce myopathies in the pectoral muscle, especially WB. This may be related to the role of the phosphagen system in maintaining energy homeostasis in the pectoral muscle, resulting in higher glycogen content and lower muscle pH.

## Figures and Tables

**Table 1:** Ingredient composition (%) and nutrient analysis of starter, grower and finisher diets.

Ingredient	Starter (0–10 Day)	Grower (10–28 Day)	Finisher (28–42 Day)
Corn	54.08	58.83	63.09
Soybean meal (48% crude protein)	35.00	30.64	25.61
Corn gluten meal (60% crude protein)	3.88	3.01	3.50
Soya oil	2.16	3.00	3.65
Limestone	1.70	1.57	1.45
Monodicalcium phosphate (21.8%)	1.07	0.91	0.75
DL-Methionine (99%)	0.31	0.28	0.25
Pellet binder	0.30	0.30	0.30
Salt (NaCl)	0.30	0.30	0.30
L-Lysine HCL (78%)	0.29	0.26	0.25
Premix ^1,2^	0.20	0.20	0.20
Mold inhibitor	0.20	0.20	0.20
Sodium bicarbonate	0.20	0.20	0.20
L-Threonine (98.5%)	0.13	0.11	0.07
Choline chloride (60%)	0.11	0.11	0.11
Coccidiostat ^3^	0.06	0.06	0.06
Phytase ^4^	0.01	0.01	0.01
Total	100	100	100
Calculated and (analyzed ^5^) nutrient content (%)			
Dry mater	89.29 (90.00)	89.27 (90.03)	89.25 (88.73)
Metabolizable energy (kcal/kg)	2950	3050	3150
Crude protein	23.81 (24.73)	21.50 (22.57)	19.64 (20.23)
Calcium	0.96	0.87	0.78
Available phosphorous	0.48	0.44	0.39
Sodium	0.17	0.17	0.17
Potassium	0.97	0.89	0.79
Chloride	0.21	0.21	0.21
Dietary electrolyte balance (mEq/kg)	261	240	215
Total lysine	1.41 (1.50)	1.27 (1.28)	1.12 (1.07)
Total methionine	0.67 (0.69)	0.61 (0.57)	0.56 (0.49)
Total methionine + cysteine	1.05 (1.08)	0.96 (0.93)	0.88 (0.82)
Total threonine	1.00 (1.08)	0.89 (0.92)	0.79 (0.79)
Total arginine	1.50 (1.64)	1.35 (1.41)	1.19 (1.23)

^1^ The T1, T2, and T3 treatment diet contained 0%, 0.06%, and 0.12% guanidinoacetic acid (Creamino^®^, minimum 96% GAA; AlzChem Trostberg GmbH, Trostberg, Germany). ^2^ Vitamins provided per kg of premix: vitamin A, 12,000,000 IU; vitamin D3, 2,400,000 IU; vitamin E, 20 mg; vitamin K, 2.45 mg; vitamin B1, 1.9 mg; vitamin B2, 4.99 mg; vitamin B6, 1.94 mg; vitamin B12, 0.02 mg; niacin, 49 mg; cal-D-Pan, 14.78 mg; biotin, 0.05 mg; folic acid, 0.98 mg; copper, 9 mg; ferrous, 38.75 mg; manganese, 60 mg; zinc, 45 mg; iodine, 0.75 mg; selenium, 1 mg; antioxidant, 2.5 mg. ^3^ Coccidiostat, salinomycin, 12%. ^4^ Quantum blue 5G^®^ at 80 g/ton to provide 500 FYT (AB-Vista) delivering 0.10% available P. ^5^ The analytical values are the averages of 3 replicates; CV% < 5%.

**Table 2:** Analyzed guanidinoacetic acid content of the dietary treatments ^1^.

Parameters	Starter			Grower			Finisher		
	T1	T2	T3	T1	T2	T3	T1	T2	T3
Calculated									
Creamino^®^, mg/kg	0	600	1200	0	600	1200	0	600	1200
Analyzed ^2^									
Creamino^®^, mg/kg	<21	613	1222	<21	572	1235	<21	560	1110
GAA, mg/kg	<20	588	1173	<20	549	1186	<20	538	1066

^1^ The T1, T2, and T3 treatment diet contained 0%, 0.06%, and 0.12% guanidinoacetic acid (Creamino^®^, minimum 96% GAA; AlzChem Trostberg GmbH, Trostberg, Germany). ^2^ Values are means of 4 samples.

**Table 3:** Effects of guanidinoacetic acid (GAA) supplementation for male broilers on growth performance up to 42 days of age.

Parameters ^2^	Days	Treatment ^1^			RMSE ^3^	*p-*Value
		T1	T2	T3		
BW (g)	10	314.67	315.45	320.42	8.13	0.141
	28	1949.15	1933.03	1902.16	51.13	0.058
	42	3145.26	3146.93	3194.32	151.34	0.637
WG (g)	0–10	272.43	273.21	278.18	8.21	0.146
	0–28	1906.91	1890.79	1859.92	51.2	0.059
	0–42	3103.02	3104.69	3152.07	154.34	0.637
FI (g)	0–10	285.60	280.33	282.32	8.25	0.245
	0–28	2501.37 ^a^	2468.97 ^b^	2390.64 ^b^	93.75	0.010
	0–42	4938.02	4790.41	4851.61	197.85	0.153
FCR (g:g)	0–10	1.048 ^a^	1.026 ^b^	1.015 ^b^	0.018	<0.0001
	0–28	1.312	1.306	1.286	0.039	0.198
	0–42	1.594 ^a^	1.544 ^b^	1.540 ^b^	0.043	0.003

^1^ T1, T2, and T3 treatment diet contained 0%, 0.06%, and 0.12% guanidinoacetic acid (Creamino^®^, minimum 96% GAA; AlzChem Trostberg GmbH, Trostberg, Germany). ^2^ BW, final body weight; WG, weight gain; FI, feed intake; FCR, feed conversion ratio. ^3^ RMSE, root mean square error. ^a,b^ Lsmeans within different superscript letters differ significantly (*p* < 0.05).

**Table 4:** Effect of guanidinoacetic acid (GAA) dietary supplementation ^1^ on carcass composition and carcass cuts.

Parameters	T1	T2	T3	RMSE ^2^	*p*-Value
*n*	56	56	56		
Live weight (kg)	3.12	3.12	3.13	0.23	0.972
Carcass weight (kg)	2.58	2.57	2.57	0.18	0.956
Carcass (%)	82.63	82.28	82.08	1.87	0.295
Breast (%)	30.14	30.07	30.51	2.14	0.504
Fillet (%)	12.80	12.65	12.88	0.99	0.462
Wing (%)	9.96	9.85	9.86	1.46	0.263
Leg (%)	31.12	31.55	31.19	0.53	0.485

^1^ T1, T2, and T3 treatment diet contained 0%, 0.06%, and 0.12% guanidinoacetic acid (Creamino^®^, minimum 96% GAA; AlzChem Trostberg GmbH, Trostberg, Germany). ^2^ RMSE, root mean square error.

**Table 5:** Effect of guanidinoacetic acid (GAA) dietary supplementation ^1^ for male broilers on wooden breast and white stripping percentage % and frequency (*n*) of each score severity at 42 days of age.

Treatments	Wooden Breast Scores				White Striping Scores			
	0	1	2	3	0	1	2	3
T1	37.5% (21)	26.8% (15)	7.1% (4) ^a,b^	28.6% (16) ^a^	30.4% (17)	44.6% (25)	23.2% (13)	1.8% (1)
T2	46.4% (26)	30.4% (17)	5.4% (3) ^b^	17.9% (10) ^a,b^	39.3% (22)	42.9% (24)	17.9% (10)	0.0% (0)
T3	44.6% (25)	26.8% (15)	19.6% (11) ^a^	8.9% (5) ^b^	37.5% (21)	50.0% (28)	12.5% (7)	0.0% (0)

^1^ T1, T2, and T3 treatment diet contained 0%, 0.06%, and 0.12% guanidinoacetic acid (Creamino^®^, minimum 96% GAA; AlzChem Trostberg GmbH, Trostberg, Germany). Data for the distribution of myopathies scores among different treatments are shown as percentages with absolute values (in parentheses). Wooden breast scores are based on a 4-point scale (3 = severe, 2 = moderate, 1 = mild and 0 = normal); white striping scores are based on a 4-point scale (3 = severe, 2 = moderate, 1 = mild and 0 = normal). (Chi^2^ tests contingency table, ^a,b^ percentage within different superscript letter differ significantly (*p* < 0.05).

**Table 6:** Effect of guanidinoacetic acid (GAA) dietary supplementation ^1^ on creatine concentration, glycogen concentration and meat quality.

Parameters	T1	T2	T3	RMSE ^4^	*p*-Value
*n* ^2,3^	56 (21)	56 (21)	56 (23)		
Creatine (mg/kg)	4315.24	4570.95	4770.87	863.16	0.224
Glycogen (mg/g)	6.44 ^b^	27.19 ^a^	31.04 ^a^	13.88	<0.0001
pH_3_	6.32 ^a^	6.26 ^b^	6.25 ^b^	0.12	0.006
pH_24_	6.16 ^a^	6.07 ^b^	6.05 ^b^	0.15	0.0002
L* (lightness)	52.88	52.88	53.19	3.94	0.893
a* (redness)	1.20	1.07	1.08	1.02	0.753
b* (yellowness)	12.25	11.60	12.27	2.61	0.312
Drip loss (%)	1.55	1.39	1.53	0.55	0.321
Cooking Loss (%)	14.37	14.76	14.97	3.80	0.709
Shear force (kg)	4.27	4.15	4.31	1.15	0.754

^1^ T1, T2, and T3 treatment diet contained 0%, 0.06%, and 0.12% guanidinoacetic acid (Creamino^®^, minimum 96% GAA; AlzChem Trostberg GmbH, Trostberg, Germany). ^2^ *n* = Sample size per treatment for meat quality. ^3^ *n* = Sample size for creatine and glycogen analyzes per treatment. ^4^ RMSE, root mean square error. ^a,b^ Lsmeans within different superscript letters differ significantly (*p* < 0.05).

**Table 7:** Carcass composition with different degree of myopathies.

Traits	Wooden Breast Score (WB)				White Striping Score (WS)			RMSE ^2^	*p*-Value	
	0	1	2	3	0	1	2		WB	WS
*n* ^1^	72	47	18	30	60	77	30			
Live weight (kg)	3.14	3.10	3.18	3.17	3.10	3.12	3.23	0.23	0.534	0.059
Carcass weight (kg)	2.58	2.55	2.64	2.62	2.56	2.58	2.65	0.17	0.194	0.079
Breast (g)	768.24 ^b^	773.86 ^b^	826.81 ^a^	800.52 ^a,b^	773.20	790.40	814.97	80.99	0.035	0.122
Fillet (g)	324.61 ^b^	324.96 ^b^	352.97 ^a^	343.75 ^a^	327.93	334.26	347.52	36.58	0.007	0.108
Carcass (%)	82.07	82.12	83.20	82.45	82.62	82.57	82.20	1.86	0.126	0.641
Breast (%)	29.78 ^b^	30.36 ^b^	31.23 ^a^	30.60 ^a,b^	30.19	30.63	30.70	2.09	0.048	0.464
Fillet (%)	12.58 ^c^	12.73 ^b,c^	13.33 ^a^	13.13 ^a,b^	12.79	12.94	13.09	0.95	0.009	0.431

^1^ *n* = The score number represents the frequency of occurrence of each score. Only one white striping, score 3 was recorded in the control group and was omitted for data accuracy. WB; 0, 1, 2, 3 = wooden breast scores are based on a 4-point scale (3 = severe, 2 = moderate, 1 = mild, and 0 = normal); WS; 0, 1, 2, 3 = white striping scores are based on a 4-point scale (3 = severe, 2 = moderate, 1 = mild, and 0 = normal). ^2^ RMSE, root mean square error. ^a,b,c^ Lsmeans within different superscript letter differ significantly (*p* < 0.05).

**Table 8:** Effect of wooden breast and white stripping on creatine content, glycogen content and meat quality.

Traits	Wooden Breast Score				White Striping Score			RMSE ^3^	*p*-Value	
	0	1	2	3	0	1	2		WB	WS
*n* ^1,2^	72 (18)	47 (18)	18 (12)	30 (17)	60 (21)	77 (29)	30 (14)			
Creatine (mg/kg)	5206.11 ^a^	4682.22 ^a,^^b^	4345.00 ^b,c^	3894.71 ^c^	4712.90	4581.38	4422.86	725.19	<0.0001	0.615
Glycogen (mg/g)	28.50	20.72	17.92	19.86	27.02	19.43	21.24	19.95	0.460	0.430
pH_3_	6.26	6.26	6.29	6.32	6.26	6.30	6.29	0.12	0.262	0.111
pH_24_	6.06 ^b^	6.08 ^b^	6.12 ^a,b^	6.18 ^a^	6.09	6.12	6.12	0.15	0.005	0.543
L*	50.84 ^c^	53.44 ^b^	55.32 ^a^	55.07 ^a,b^	53.31	54.54	53.15	3.47	<0.0001	0.063
a*	1.23	1.08	1.36	1.07	1.09	1.02	1.44	1.02	0.718	0.209
b*	10.99 ^b^	12.57 ^a^	13.09 ^a^	13.12 ^a^	12.52	12.39	12.42	2.47	0.0002	0.957
Drip loss (%)	1.46	1.43	1.54	1.74	1.32 ^b^	1.68 ^a^	1.24 ^b^	0.43	0.183	<0.0001
Cooking Loss (%)	13.64 ^b^	15.86 ^a^	16.29 ^a^	15.72 ^a^	15.24	14.67	16.22	3.59	0.003	0.162
Shear force (kg)	4.52 ^a,b^	4.55 ^a^	4.09 ^a,b^	3.54 ^b^	4.06	4.03	4.45	1.11	0.001	0.251
rHard (N/cm^2^)	29.95 ^b,c^	27.12 ^c^	37.30 ^a,b^	48.62 ^a^	31.58	32.86	38.31	18.18	0.0002	0.325
cHard (N/cm^2^)	46.20	47.17	38.67	41.52	39.31 ^b^	41.56 ^b^	49.30 ^a^	13.57	0.079	0.014

^1^ *n* = The scores number represents the frequency of occurrence of each score. Only one white striping, score 3 was recorded in the control group and was omitted for data accuracy. WB; 0, 1, 2, 3 = wooden breast scores are based on a 4-point scale (3 = severe, 2 = moderate, 1 = mild, and 0 = normal); WS; 0, 1, 2, 3 = white striping scores are based on a 4-point scale (3 = severe, 2 = moderate, 1 = mild, and 0 = normal). ^2^ *n* = Sample size for creatine and glycogen analyzes per myopathies scores. ^3^ RMSE, root mean square error. L* (lightness), a* (redness), b* (yellowness), cHard (cooked meat hardness), and rHard (raw meat hardness). ^a,b,c^ Lsmeans within different superscript letter differ significantly (*p* < 0.05).

## Data Availability

Data sharing not applicable.
